# Inguinal Hernia Mesh Repair: The Factors to Consider When Deciding Between Open Versus Laparoscopic Repair

**DOI:** 10.7759/cureus.19628

**Published:** 2021-11-16

**Authors:** Sri Vallabh Reddy Gudigopuram, Ciri C Raguthu, Harini Gajjela, Iljena Kela, Chandra L Kakarala, Mohammad Hassan, Rishab Belavadi, Ibrahim Sange

**Affiliations:** 1 Research, Our Lady of Fatima University College of Medicine, Metro Manila, PHL; 2 Research, Tianjin Medical University, Tianjin, CHN; 3 Family Medicine, Jagiellonian University Medical College, Krakow, POL; 4 Internal Medicine, Jawaharlal Institute of Post-Graduate Medical Education and Research (JIPMER), Pondicherry, IND; 5 Internal Medicine, Mohiuddin Islamic Medical College, Mirpur, PAK; 6 Surgery, Jawaharlal Institute of Post-Graduate Medical Education and Research (JIPMER), Pondicherry, IND; 7 Research, California Institute of Behavioral Neurosciences & Psychology, Fairfield, USA; 8 Research, K. J. Somaiya Medical College, Mumbai, IND

**Keywords:** chronic pain, recurrence rate, outcomes, laparoscopic inguinal hernia repair, open inguinal hernia repair

## Abstract

Inguinal hernia repair is one of the most commonly performed surgical procedures worldwide. An inguinal hernia occurs due to a defect in the abdominal wall, which allows the abdominal contents to pass through it. Although the placement of mesh over the defect is the gold standard to close the defect, there are various approaches to achieving it, out of which two of the most widely accepted techniques are laparoscopic inguinal hernia repair (LIHR) and open inguinal hernia repair (OIHR). However, the approach of choice widely fluctuates with regards to various factors such as patient history, type of hernias, and surgeons' preference. It is imperative to understand the variations in outcomes of different approaches and how best they fit an individual patient in deciding the technique to be undertaken. This article has reviewed many studies and compared the two techniques in terms of chronic pain, the time required to return to activity, rate of recurrence, and cost-effectiveness.

## Introduction and background

Groin hernias arise from a defect in the abdominal wall and consist of inguinal and femoral hernias [1]. The lifetime risk of acquiring a groin hernia is 27% for men and 3% for women [2]. The risk escalates with age, and the frequency of hernia repair increases from 0.25% at age 18 years to 4.2% at age 75 to 80 years [2]. Inguinal hernias could be of predominantly two types - direct or indirect based on their site of herniation to the inferior epigastric vessels (IEV) and Hesselsbach's triangle [3]. More than 90% of all inguinal hernias occur in males. Femoral hernias are noticeably more common in females compared to males; however, less common than inguinal hernias [4]. The risk factors for an inguinal hernia include the family history of groin hernia, chronic obstructive pulmonary disease, smoking, low body-mass index, increased intraabdominal pressure, collagen diseases, patent processus vaginalis, history of appendectomy, and peritoneal dialysis [5]. Although inguinal hernias usually present as an asymptomatic bulge in the groin, patients can occasionally present with symptoms such as groin pain that worsens toward the end of the day, an increase in the size of the bulge, and a dragging sensation in the groin [6]. A comprehensive history of diet, lifestyle, and comorbidities, along with a detailed physical examination, is reliable enough to conclude the diagnosis of an inguinal hernia [6]. However, further diagnostic tests are often required in challenging cases, such as occult hernias or hernias in female patients [6,7]. The first line imaging modality used is ultrasonography (USG), which helps diagnose suspected groin hernias that are not clinically evident [8,9]. A magnetic resonance imaging (MRI) with Valsalva maneuver should be performed if the clinical suspicion is high despite negative USG findings. MRI is superior to a computed tomography scan (CT-scan) and USG in diagnosing hernias [10]. In some patients, herniography can be used, which is superior to USG and CT-scan [9]. Watchful waiting is the appropriate and safe option for men whose daily life activities are not affected and have a reducible hernia [11]. But, hernia repair must be considered when patients present with pain because there is an increased chance of incarceration or strangulation, especially for femoral hernias due to the rigid borders of the femoral canal [3,12]. There are two different routes to repair an inguinal hernia, open and laparoscopic mesh repairs. Any of the two aforementioned approaches can be preferred by a general surgeon to repair an inguinal hernia and can vary according to various factors such as patient profile, operating time, complication rate, etc. This article aims to highlight the features of laparoscopic and open repair approaches for hernia repair, and compare and contrast these two approaches regarding elements like patient profile, complication rate, recurrence rate, etc.

## Review

The types of groin hernias are defined based on their anatomic relation to the surrounding structures. Direct inguinal hernias occur due to laxity of the inguinal canal floor and present above the inguinal ligament, medial to the IEV, and within the Hesselbach's space. Indirect inguinal hernias usually occur due to the forced opening of Processus Vaginalis by increased intraabdominal pressure and present above the inguinal ligament lateral to the IEV and outside of the Hesselbach's triangle [3,13]. Femoral hernias occur due to weakness in the femoral canal and present below the inguinal ligament and medial to femoral vessels [13]. The establishment of mesh in the late 1960s has transformed the repair of groin hernias. Since then, multiple approaches to repair the defect have been described. They include tension-free mesh repair via the open inguinal hernia repair (OIHR) and the laparoscopic inguinal hernia repair (LIHR) [3,14]. The success of an inguinal hernia repair depends on several factors such as hernia recurrence, infection rate, neuralgia, and the rate of complications [15].

Laparoscopic inguinal hernia mesh repair 

The first LIHR was introduced by Ralph Ger and colleagues in 1982 and gained popularity in late 1990. They had performed on dogs by repairing the patent processus vaginalis [16]. The laparoscopic approaches are primarily of two types - transabdominal preperitoneal repair (TAPP) and total extraperitoneal repair (TEP) [17]. In 1992, Arregui and colleagues first introduced the TAPP approach, and the placement of mesh in this approach requires access into the peritoneal cavity [18]. In 1993, McKernan and colleagues first performed the TEP approach, which requires preperitoneal dissection and mesh placement without entering the abdominal cavity [19]. As per the latest literature, the outcomes in terms of postoperative pain, overall complications, and recurrence rates were similar in both TAPP and TEP. Hence, the choice of technique depends upon the surgeon’s skill, preference, and experience [20,21]. The overall advantages of the laparoscopic approach are as follows [3,22,23].

· Repair of recurrent inguinal hernias.

· Minimal post-operative and chronic pain.

· Earlier return to daily life activities.

· Repair of bilateral inguinal hernias without an increase in morbidity.

The laparoscopic approach is very beneficial in the repair of recurrent inguinal hernias that have been previously repaired with a traditional open anterior approach as the laparoscopic posterior approach avoids the significant scar tissue and allows the surgeon to approach from a previously untouched space [3,20]. LIHR has shown a decrease in chronic pain and the time required to return to full activity when performed in recurrent inguinal hernia patients [23]. Pisanu et al. conducted a meta-analysis in 2015, which included seven studies comparing laparoscopic and open (Lichtenstein) approaches. A total of 647 patients with recurrent inguinal hernias were randomized to undergo either technique. Among them, 333 underwent the Laparoscopic (TAPP/TEP) technique and 314 open (Lichtenstein) technique. The analysis revealed that 9.2% of patients in the laparoscopic group and 21.5% in the open group complained of chronic pain. At the same time, the patients in the laparoscopic group required 13.9 days and patients in the open group 18.4 days to return to work. These findings concluded that laparoscopic repair reduces chronic pain and the time required to return to work [24].

Bilateral inguinal hernias can occur in approximately 8% to 30% of patients in whom LIHR has proved beneficial in decreasing the recurrence and the risk of complications associated with it [20,25]. A study conducted in 2013 evaluated and compared the treatment outcomes of the bilateral inguinal hernia repair by a laparoscopic (TEP) approach and open (Lichtenstein) approach techniques. A total of 325 patients with bilateral inguinal hernias hospitalized at that institution between 2006 and 2011 were analyzed based on their records. Among them, 234 cases underwent a laparoscopic approach, and 91 patients underwent an open approach. The analysis showed complications in 2.5% of the cases in the laparoscopic group and 27.4% cases in the open group. The study stated that the laparoscopic approach had a 10-fold decrease in complications and morbidity than the open repair and concluded that laparoscopic repair is the gold standard for bilateral inguinal hernias [26].

LIHR also boasts an optimistic postoperative course by decreasing the incidence of post-surgical chronic pain and the time required to return to work [27,28]. Eklund et al. performed a randomized multi-center study with a five-year follow-up were performed in 2010 on a group of adult men with primary inguinal hernia. The study compared the frequency of chronic postoperative pain categorized into mild, moderate, or severe after the laparoscopic (TEP) repair and open (Lichtenstein) repair. Of 1,370 out of 1,512 randomized patients, 665 underwent TEP, and 705 underwent an open repair. With the results of 1.9% in the TEP group and 3.5% in the open group with moderate to severe pain after five years, the study concluded that laparoscopic hernia repair had been associated with less chronic pain than an open hernia repair [28]. The above-mentioned study also assessed the short-term complications, reoperations, postoperative pain, and time to resumption to normal activity after primary inguinal hernia repair in both laparoscopic and open groups. It concluded that the laparoscopic (TEP) group returned to normal activities earlier than the open group [29].

However, LIHR has some limitations with regards to the recurrence rate as it was significantly higher after laparoscopic repair than open repair in primary hernias [30]. A randomized study was done on men above 18 years of age with an inguinal hernia at 14 veterans’ affairs (VA) medical centers. The primary outcome of the study was a recurrence of inguinal hernia at a two-year follow-up. A total of 2,164 patients were randomized, out of whom 1,983 underwent surgery, and a two-year follow-up was completed in 1,696 patients. Among them, 862 underwent laparoscopic approach and 834 underwent open approach. The study concluded that the recurrence was more common in the laparoscopic group (10.1%) than in the open group (4.9%) [30]. These studies on LIHR are summarized in Table *1*.

**Table 1:** Summary of included studies regarding the outcomes of laparoscopic inguinal hernia repair. TRRDA, Time Required to Return to Daily Activities; VA, Veterans Affairs; LIHR, Laparoscopic inguinal hernia repair; OIHR, Open Inguinal Hernia Repair.

STUDY	DESIGN	SUBJECTS	STUDY OBJECTIVES	RESULTS	CONCLUSION
Pisanu et al. (2015) [24]	Meta-analysis	Adults with recurrent inguinal hernias.	To assess the benefits of LIHR in recurrent inguinal hernia patients.	In the LIHR group, 9.2% had chronic pain and The TRRDA was 13.9 days. In the OIHR group, 21.5% had chronic pain and the TRRDA was 18.4 days.	The laparoscopic technique had more benefits over the open repair for recurrent inguinal hernias.
Timisescu et al. (2013) [26]	Analysis of records from a single institution	Cases with an intraoperative diagnosis of bilateral inguinal hernia	Compare and analyze the complications and morbidity in bilateral inguinal hernia repair.	2.5% of the population in the LIHR group and 27.4 of the population in the OIHR group had complications.	The laparoscopic approach is the gold standard for bilateral inguinal hernia.
Eklund et al. (2010) [27]	Randomized multicenter study	Adult men with primary inguinal hernia	Chronic pain is categorized into mild, moderate, or severe at a 5-year follow-up.	1.9% of the population in the LIHR group and 3.5% of the population in the OIHR group had reported moderate/severe pain.	LIHR leads to minimal chronic pain compared to an OIHR.
Eklund (2006) [28]	Prospective randomized study	Patients undergoing primary inguinal hernia repair	A number of days needed to return to work postoperatively.	The TRRDA was 7 days in the LIHR group and 12 days in the OIHR group.	The LIHR group returned earlier to work than The OIHR group.
Neumayer et al. (2004) [29]	Randomized study	Men with an inguinal hernia at 14 VA medical centers.	Recurrence of hernia at two-year follow-up.	The recurrence rate was 10.1% in the LIHR group and 4.9% in the OIHR group.	LIHR group had more recurrences than the OIHR group.

Another drawback of laparoscopic repair is the lengthy operative time that the surgeon usually requires to carry out the procedure [24]. The use of general anesthesia as a part of laparoscopic surgery restricts patients with cardiac or pulmonary co-morbidities from undergoing the procedure relative to the open approach, which can be performed under local anesthesia [3,23]. A prospective randomized study performed in 2003 studied 25 patients in the open group and 25 patients in the TEP group. The study had revealed that the laparoscopic (TEP) repair took 75.72 +/- 31.6 min, which was significantly longer than the open repair, which took 54 +/- 15 min to perform the procedure [31]. However, in the analysis between TAPP and TEP, the TEP technique had a longer operative time due to its smaller intraoperative field and increased risk of bleeding while dissecting the pre-peritoneal space. For this reason, the learning curve is much higher for the TEP technique than the TAPP technique. But, at the same time, the TAPP approach is associated with abdominal organ injury due to its wide intraoperative field [32]. Hence, commonly the laparoscopic procedures are associated with an increased risk of complications such as hemorrhage, bowel, bladder, and vascular injuries [33].

According to a review of the latest research studies, certain outcomes such as recurrence were likely influenced by subjective factors such as experience and the skills of the operating surgeon [34]. Compared to the open approach, the learning curve for the laparoscopic repairs is around 50-100 cases, which is more cumbersome to master [27]. In terms of operating time in the TEP approach, a surgeon needs a minimum of 50 cases to reach the plateau and 60 cases or more to limit the complications, recurrence, and conversion rate [35]. A population-based retrospective cohort study performed in Canada identified 93,501 adults using linked administrative databases who underwent primary inguinal hernia repair (85.4% open approach vs. 14.6% laparoscopic approach) with a 5.5-year median follow-up. First, the study revealed that the five-year overall risk for recurrent inguinal hernia repair (IHR) was 2.0% in the open group and 3.4% in the laparoscopic group. But after adjusting the patient and surgeon factors, the study found that the risk for recurrent inguinal hernia repair in patients who had surgery done by a surgeon with an experience of >50 cases/year in the previous year was less [36]. Therefore, despite its limitations, The surgeon’s good skill and experience with the procedure can limit them. The laparoscopic approach continues to be preferred by surgeons with respect to a flexible patient profile due to its improving and upcoming methodologies [24,37].

Open inguinal hernia mesh repair

The OIHR approach is the most commonly performed technique, with the primary basis of each technique being either pure tissue repair or mesh repair [38]. The pure tissue repair is further classified into different techniques based on the approach, the open anterior (Bassini, Shouldice, Mcvay repairs) and the open posterior (Nyhus iliopubic tract repair) [3]. The Bassini repair was first introduced in the late 1800s and this procedure included reconstructing the abdominal wall by suturing the internal oblique, the transversus muscle, and the transversalis fascia to the inguinal ligament with the help of interrupted permanent sutures [39]. The McVay hernia repair is similar to the Bassini repair except that the three layers in Bassini repair are sutured to the cooper’s ligament rather than the inguinal ligament [39]. In 1952, Earl Shouldice introduced the Shouldice repair, which is a modification of the Bassini repair where the posterior wall of the inguinal canal is strengthened with four layers of fascia and aponeuroses of the oblique muscles with continuous running suture [40]. The main objective of any hernia repair is to correct the transversus abdominis layer to normal, which was achieved by Nyhus using the posterior preperitoneal approach and iliopubic tract repair [41]. But unfortunately, all these techniques had portrayed problems regarding the tension created by sutures on the surrounding tissues, leading to recurrence [3]. In 1958, Usher et al. introduced a technique to repair the hernia using marlex mesh, which had the benefit of freeing the tension on the surrounding tissues [42]. However, the use of mesh repair was not widespread until 1984, when Lichtenstein first coined the term “tension-free” repair [43]. Since then, the Lichtenstein open mesh repair has been the most widely accepted open hernia repair because of its usefulness in any patient with any hernia. It also had a short learning curve and reliable outcomes with a low recurrence rate than other open tissue repair techniques [14,44].

Many studies were performed to prove the effectiveness of Lichtenstein over other tissue repairs. One such study was done in 2002, which gathered information from electronic databases. It had conducted 62 relevant comparisons in 58 trials which included 11,174 participants. Among them, 6,901 had individual patient data, 2,390 had supplementary aggregated data, and 1,883 had published data. The analysis showed that 88 in 4,426 of the mesh repair vs. 187 in 3,795 of the non-mesh repair had a hernia recurrence, and 120 in 2,368 in the mesh group vs. 215 in 1,998 in the non-mesh group had persistent pain concluding that the mesh repair is associated with a low recurrence rate and less persistent pain than the non-mesh repair [45].

One of the main reasons for the wide acceptance of Lichtenstein repair over laparoscopic repair worldwide is because it can be easily taught to the trainees with the same reproducible results as the senior surgeons [46]. Merola et al. performed a retrospective analysis to determine the required number of cases to achieve the learning curve for the Lichtenstein procedure. They compared the outcomes of the first 100 procedures performed by four trainees from three different hospitals to the same number of procedures performed by three senior surgeons. Based on the evaluation, they found that an average of 37 to 42 procedures is required for a trainee to reach the level of a senior surgeon [47].

OIHR also has benefits in terms of operative time, use of local anesthesia, and cost-effectiveness [44].

Dhankar et al. performed a prospective randomized trial to compare the outcomes of LIHR and OIHR in terms of anesthesia and cost-effectiveness. The study recruited 72 patients, among whom 36 patients were randomized to the LIHR group and 36 to the OIHR group. After performing a per-protocol analysis, the study concluded that the OIHR had a shorter operating time and lower cost to anesthetics. It stated that open repair would be the best option to perform in developing nations, as long-term patients’ comfort was equal to laparoscopic repair [48].

The same findings can be paired with a study performed by Schneider et al., which aimed to analyze and compare the costs of laparoscopic repair and open Lichtenstein repair. They reviewed multiple cases performed at two hospitals and concluded that the laparoscopic procedure would cost 852 US dollars more than the open Lichtenstein repair [49].

But, when compared to laparoscopic repair (TAPP and TEP), the open Lichtenstein repair has a drawback concerning chronic postoperative pain and the time required to return to work [50]. A systematic review and meta-analysis of Randomized control trials were conducted in 2021 to evaluate the effective inguinal hernia approaches. The study included thirty-five trials consisting of 7,777 patients. Among them, 3,496 underwent Lichtenstein repair, 1,296 underwent TAPP, and 3,012 underwent TEP repair. The analysis revealed that the laparoscopic patients had less postoperative pain according to the visual analog scale. In terms of postoperative chronic pain, the TAPP and TEP had an RR=0.36 and 0.36, respectively, vs. the Lichtenstein repair. Regarding the time required to return to work, the weighted mean difference among TAPP vs. Lichtenstein was -3.3, and TEP vs. Lichtenstein was -3.6. Therefore, they concluded that the Lichtenstein was inferior to laparoscopic among the outcomes mentioned above [50]. As mentioned previously, hernia recurrence is a chronic complication, and it is the main determining factor for a successful hernia repair, and the rate of recurrence is also a beneficial outcome in judging a technique [51]. Sevinc et al. conducted a prospective randomized study in 2019 to compare the postoperative outcomes of the LIHR and the OIHR. It included 302 patients with an inguinal hernia, 147 underwent TEP repair, and 155 underwent OIHR. After a mean follow-up for 40.95 months, the recurrence rate was similar in both groups [52].

Although the open approach has some drawbacks in terms of chronic pain and recovery time, the overall complication rate was almost equal to the laparoscopic approach [53]. Kargar et al. performed a randomized study that aimed to compare the short-term complications of LIHR and Lichtenstein repair. The study included 120 patients who underwent inguinal hernia repair, among whom 60 were randomized to LIHR and 60 into OIHR. The subjects were followed for six weeks and assessed for hematoma/seroma, urinary retention, and wound infection during the hospital stay. The study showed that the differences in these outcomes among the two groups were not statistically significant and hence concluded that both approaches have no difference in the rate of these complications [54]. The articles on OIHR are summarized in Table *2*.

**Table 2:** Summary of included studies regarding the outcomes of open inguinal hernia repair. LIHR, Laparoscopic Inguinal Hernia Repair; OIHR, Open Inguinal Hernia Repair; TRRDA, Time Required to Return to Daily Activities.

STUDY	DESIGN	SUBJECTS	STUDY OBJECTIVES	RESULTS	CONCLUSION
Dhankar et al. (2014) [48]	Prospective randomized trial	72 patients with inguinal hernia.	To compare the open approach and laparoscopic approach in terms of anesthesia, operative time, and cost.	The open approach had a shorter operating time, usage of local anesthetic in the open approach is associated with lesser cost, and patients in both groups had the same level of comfort in the long term.	Due to the low cost of anesthetics, same long-term comfort of patients it is recommended that open repair would be best in resource-scarce countries.
Aiolfi et al. (2021) [50]	Meta-analysis	Thirty-five trials consisted of a total of 7,777 patients with inguinal hernia.	Evaluate and compare the laparoscopic and open approaches	The chronic pain and TRRDA were less in the LIHR group than the OIHR group.	The Lichtenstein approach was inferior to the laparoscopic approach.
Sevinc et al. (2019) [52]	Prospective randomized study	302 adult patients with inguinal hernia.	To compare postoperative outcomes in Open and Laparoscopic approaches.	After a mean follow-up of 40.95 months, both groups had a similar recurrence rate.	The open approach and laparoscopic approach had the same recurrence rate.
Kargar et al. (2015) [54]	Randomized trial	120 patients with inguinal hernia.	To compare short-term complications of the open approach and the Laparoscopic approach.	After a follow-up of 6-weeks, the rate of hematoma/seroma, urinary retention, and wound infections were not statistically significant.	There is no difference in the rate of immediate complications between the open approach and the laparoscopic approach.

Therefore, when performed by training residents, the outcomes of the OIHR are almost equal to the experienced surgeon. The technique’s feasibility, easy learning curve, and cost-effectiveness make it the most widely accepted approach to repair a hernia [44].

Limitations

The article discusses only the generalized overview of the outcomes mentioned above. It does not acknowledge the specific factors as to why those outcomes occur. Another major limitation is that it included studies of inguinal hernia only on men but did not include female patients and pediatric patients.

## Conclusions

Today every conventional technique is shifting towards a modern, minimally invasive technique with Inguinal hernia repair being one of them. In this article, we have referred to multiple studies to describe the fate of outcomes such as postoperative pain, chronic pain, the time required to return to normal activities, and complications with regards to the open approach and the laparoscopic approach. This article gives a generalized overview of the aforementioned outcomes and helps surgeons decide their approach when a patient presents with an inguinal hernia. The patients who underwent laparoscopic repair have a less chronic postoperative and earlier return to normal activities, unlike those who underwent open repair. These favorable outcomes can offset the high cost of this procedure and make it a good option for bilateral inguinal hernias, femoral hernia, and recurrent hernias despite being limited by a high recurrence rate and a longer operation time. Open repair's easy learning curve, use of local anesthesia, and cost-effectiveness make it a widely accepted procedure worldwide and suitable for primary inguinal hernias despite having drawbacks such as chronic pain and an increase in the time required to return to work. In summary, both techniques have their benefits, and a surgeon must decide the type of approach based on their expertise and individual patient's favored outcome. The clinical implication of this review article is to determine how these various outcomes differ with respect to the type of approach and how a surgeon could tailor them based on their experience and the patient profile. As many other factors also determine these outcomes, we recommend that more future studies need to be performed to understand these differences between the techniques.
